# Supplementary material for: The genome sequence of the German scorpionfly, Panorpa germanica Linnaeus, 1758
Source: Wellcome Open Res. 2024 May 21;9:285. [Version 1] doi: 10.12688/wellcomeopenres.22259.1 (PMC11464959; doi:10.12688/wellcomeopenres.22259.1)
Supplement: Peer review history

# Reviewer report: Reviewer response for version 1

**DOI:** 10.21956/wellcomeopenres.24536.r100447

**Published:** 2024-10-09

**Authors:** Lianxi Xing

**Affiliations:** 1 Northwest University, Xi’an, China

The Darwin Tree of Life Project has significantly advanced entomological research by providing comprehensive genomic data on a wide array of insect species. This project has facilitated deeper insights into insect biodiversity and evolutionary relationships. Especially for Mecoptera, which have historically lacked genomic data, the project offers crucial insights and fills critical gaps in our understanding.

 I have a few questions about this article:

  

1. Several *Panorpa* genomes have been reported so far, and it would be nice if they were mentioned in Background.
2. The samples used for Hi-c and Pacbio sequencing seem to be different specimen. Why treat it that way, not enough tissue? I remember using the same specimen in an earlier article.
3. In Fig. 5, what might be the reason for the relatively weak interactions between the first two chromosomes?

Overall, this has been a very rewarding work, and I am greatly expecting to see the genomes of families such as Boreidae, Nannochoristidae, etc. published in the future.

Are sufficient details of methods and materials provided to allow replication by others?

Yes

Is the rationale for creating the dataset(s) clearly described?

Yes

Are the datasets clearly presented in a useable and accessible format?

Yes

Are the protocols appropriate and is the work technically sound?

Yes

Reviewer Expertise:

Insect Systematic Entomology and Genomics

I confirm that I have read this submission and believe that I have an appropriate level of expertise to confirm that it is of an acceptable scientific standard.

# Reviewer report: Reviewer response for version 1

**DOI:** 10.21956/wellcomeopenres.24536.r100439

**Published:** 2024-10-08

**Authors:** Bao-Zhen Hua

**Affiliations:** 1 Northwest A&F University, Yangling, Shaanxi, China

The manuscript deals with the genome sequence of the German scorpionfly, *Panorpa germanica *Linnaeus, 1758, and is a great contribution to the study of insects, especially the order Mecoptera. In general, the manuscript is well written and suitable for indexing. Therefore, I recommend to indexing this paper in this journal.

 I have conducted the taxonomy of Panorpidae for almost three decades, but I have never found that the female scorpionflies have such an organ as ovipositor. Please read related literature to use more specific words.

 In the Methods, the authors mentioned that a male specimen was collected and used for genome sequencing. In the first paragraph of page 5, “the specimen… was identified by Duncan Sivell. The specimen was preserved in liquid nitogen”. In the second paragraph, “The specimen was… identified by Liam Crowley and preserved on dry ice.” Please explain the contradictions.

Are sufficient details of methods and materials provided to allow replication by others?

Yes

Is the rationale for creating the dataset(s) clearly described?

Yes

Are the datasets clearly presented in a useable and accessible format?

Yes

Are the protocols appropriate and is the work technically sound?

Yes

Reviewer Expertise:

taxonomy, morphology, and phylogeny of Mecoptera

I confirm that I have read this submission and believe that I have an appropriate level of expertise to confirm that it is of an acceptable scientific standard.
